# Physiological Validation of an Airborne Ultrasound Based Surface Motion Camera for a Contactless Characterization of Breathing Pattern in Humans

**DOI:** 10.3389/fphys.2019.00680

**Published:** 2019-05-29

**Authors:** Marie-Cécile Niérat, Pierantonio Laveneziana, Bruno-Pierre Dubé, Pavel Shirkovskiy, Ros-Kiri Ing, Thomas Similowski

**Affiliations:** ^1^INSERM, UMRS1158 Neurophysiologie Respiratoire Expérimentale et Clinique, Sorbonne Université, Paris, France; ^2^Assistance Publique – Hôpitaux de Paris (AP-HP), Groupe Hospitalier Pitié-Salpêtrière Charles Foix, Service des Explorations Fonctionnelles de la Respiration, de l’Exercice et de la Dyspnée, Département R3S, Paris, France; ^3^Carrefour de l’Innovation et de l’Évaluation en Santé, Centre de Recherche du Centre Hospitalier de l’Université de Montréal, Montreal, QC, Canada; ^4^Institut Langevin, CNRS UMR7587, ESPCI ParisTech, PSL Research University, Paris, France; ^5^Assistance Publique – Hôpitaux de Paris (AP-HP), Groupe Hospitalier Pitié-Salpêtrière Charles Foix, Service de Pneumologie, Médecine Intensive et Réanimation, Département R3S, Paris, France

**Keywords:** breathing pattern, breathing variability, observer effect, vibrometry, airborne ultrasound, contactless breathing measurement

## Abstract

Characterizing the breathing pattern in naturally breathing humans brings important information on respiratory mechanics, respiratory muscle, and breathing control. However, measuring breathing modifies breathing (observer effect) through the effects of instrumentation and awareness: measuring human breathing under true ecological conditions is currently impossible. This study tested the hypothesis that non-contact vibrometry using airborne ultrasound (SONAR) could measure breathing movements in a contactless and invisible manner. Thus, first, we evaluated the validity of SONAR measurements by testing their interchangeability with pneumotachograph (PNT) measurements obtained at the same time. We also aimed at evaluating the observer effect by comparing breathing variability obtained by SONAR versus SONAR-PNT measurements. Twenty-three healthy subjects (12 men and 11 women; mean age 33 years – range: 20–54) were studied during resting breathing while sitting on a chair. Breathing activity was described in terms of ventilatory flow measured using a PNT and, either simultaneously or sequentially, with a SONAR device measuring the velocity of the surface motion of the chest wall. SONAR was focused either anteriorly on the xiphoid process or posteriorly on the lower part of the costal margin. Discrete ventilatory temporal and volume variables and their coefficients of variability were calculated from the flow signal (PNT) and the velocity signal (SONAR) and tested for interchangeability (Passing-Bablok regression). Tidal volume (VT) and displacement were linearly related. Breathing frequency (BF), total cycle time (TT), inspiratory time (TI), and expiratory time (TE) met interchangeability criteria. Their coefficients of variation were not statistically significantly different with PNT and SONAR-only. This was true for both the anterior and the posterior SONAR measurements. Non-contact vibrometry using airborne ultrasound is a valid tool for measuring resting breathing pattern.

## Introduction

Characterizing breathing pattern is one of the fundamental objectives of respiratory physiology and respiratory medicine. This is true at rest (tidal breathing) and during exercise, in health and in disease. Indeed, the breathing pattern of an individual integrates information pertaining to respiratory mechanics, respiratory muscles, and breathing control. It can be analyzed in terms of global indices such as breathing frequency (BF) and tidal volume (VT). It can also be analyzed in terms of the temporal decomposition of the ventilatory cycle (period or total cycle time, TT divided in inspiratory time TI and expiratory time TE) (Milic-Emili and Grunstein, 1976) giving access to composite indices pertinent to breathing control (mean inspiratory flow, VT/TI, reflecting the intensity of the neural drive to breathe) and respiratory muscle loading (duty cycle TI/TT) (Milic-Emili and Grunstein, 1976). The breathing pattern can also be analyzed in terms of the variability of the above variables. Respiratory variability relates to breathing control (Fiamma et al., 2007b) and the load-capacity balance of the respiratory system (Schmidt et al., 2010) defined as the relationship that exists between the mechanical impedance of the respiratory system (load) and the respiratory muscle strength available to overcome this impedance to produce ventilation (capacity). Likewise, improving the load-capacity balance by providing mechanical ventilatory support to patients suffering from acute respiratory failure increases respiratory variability (Schmidt et al., 2010). Respiratory variability is also linked to clinical prognosis. A decreased breath-to-breath variability of tidal volume has thus been shown to predict failure from ventilator weaning in critically ill patients (Wysocki et al., 2006).

Instruments describing human ventilatory activity have been developed since the nineteenth century (Marey, 1864; Michaelis, 1966). Many measure thoracoabdominal displacements or changes in shape and dimension. These include early “spirographs” consisting in air-filled or water-filled tubings placed around the body and connected to pressure transducers; magnetometers, measuring the linear distance between two electromagnets placed in front of each other across the body (Mead et al., 1967); respiratory inductive plethysmography, measuring the self-inductance variations produced in sinusoid wire coils by the respiratory movements (Cohn et al., 1982); optoelectronic plethysmography, reconstructing changes in lung volume through motion analysis of the three-dimensional coordinates of reflective markers placed on the thoracoabdominal surface (Cala et al., 1996; Aliverti et al., 2001); structured light plethysmography, reconstructing changes in lung volumes through the stereoscopic analysis of the distortions that these changes induce in a checkerboard pattern projected on the chest of an individual (Gourlay et al., 1984; Peacock et al., 1984; Drummond and Duffy, 2001; Elshafie et al., 2016; Hmeidi et al., 2017; Motamedi-Fakhr et al., 2017a,b; Nierat et al., 2017). Other devices assess ventilatory activity through measures of the quantity of inspired air and expired gas that it mobilizes. These include spirometers (direct measurement of displaced volumes), body plethysmographs (indirect measurements of displaced volumes through the corresponding changes in pressure in a closed chamber), and pneumotachographs (evaluation of flow through the drop in pressure induced by breathing in a laminarized conduit). With the notable exception of structured light plethysmography, all these instruments require some degree of contact with the body. Without exception, all these instruments imply awareness of the measurement by the subject.

Yet human respiratory behavior is highly sensitive to both instrumentation and observation. In other words, measuring breathing changes breathing, a phenomenon referred to as “observer effect.” For example, the use of a mouthpiece to measure ventilatory flow with a pneumotachograph introduces a major perturbation to breathing. Several studies have demonstrated patent changes in breathing pattern induced by this type of measurements (Gilbert et al., 1972; Askanazi et al., 1980; Weissman et al., 1984; Perez and Tobin, 1985; Fiamma et al., 2007a; Rameckers et al., 2007; Wagner and Clark, 2016). It has recently been shown that breathing through a pneumotachograph was sufficient to alter breathing variability (Nierat et al., 2017). This “instrumental observer effect” is most probably of smaller amplitude with surface devices than with devices requiring a mouthpiece (and possibly negligible with structured light plethysmography). Nevertheless, the cognitive and emotional and cognitive impacts of being observed are bound to induce breathing modifications (Western and Patrick, 1988; Han et al., 1997). As a result, measuring human breathing under true ecological conditions is currently impossible.

To achieve such a measure, a contactless device is necessary. In addition, this device should not resort to any visible or audible interaction with the subject. Non-contact vibrometry using airborne ultrasound theoretically meets these requirements. Based on existing data showing that this technology (hereafter named SONAR for practical purposes) can detect heartbeats (Jeger-Madiot et al., 2017), the present study was designed and conducted to test the hypothesis that SONAR-derived measurements of chest wall displacement are valid (namely interchangeable with reference pneumotachograph measurements) and can detect the observer effect linked to PNT’s use.

## Materials and Methods

### Subjects

Twenty-three healthy subjects with no past history of pulmonary or neuromuscular disease participated in the study (12 men and 11 women; mean age 33 years – range: 20–54, height 172 ± 9.6 cm, weight 68.7 ± 15.6 kg, body mass index 23 ± 3.7 kg/m^2^, xyphoidal circumference 86.3 ± 14.4 cm).

The study conformed to the Declaration of Helsinki and was approved by the legal and ethical French authorities (Comité de Protection des Personnes Ile-de-France VI Pitié-Salpêtrière, Paris, France). The subjects received detailed information about the methods used and the protocol, and provided written consent.

### Respiratory Signals

Breathing activity was described in terms of ventilatory flow (l min^-1^) as measured using a heated low-resistance pneumotachograph (PNT) (Hans Rudolf, 3700 series, linearity range 0–160 l/min; Kansas City, MO, United States) connected to a linear differential pressure transducer (±5 cm H20, Validyne, Northridge, CA, United States). Breathing activity was also described, either simultaneously or sequentially, with a non-contact vibrometer using airborne ultrasound (SONAR) measuring the velocity (mm s^-1^) of the surface motion of the chest wall (see detailed description below). The PNT and the SONAR signals were digitized using a Powerlab System (AD Instruments, Castle Hill, Australia) and analyzed using the Chart software (Chart 7.3; AD Instruments, Castle Hill, NSW, Australia).

The following variables were measured from both types of signals: breathing frequency (Bf), inspiratory time (TI), expiratory time (TE), total cycle time (TT). Tidal volume (VT) was obtained from electrical integration of the PNT flow signal. Chest wall displacement was obtained from integration of the SONAR velocity signal.

### Non-contact Vibrometer Using Airborne Ultrasound (SONAR)

**FIGURE 1:**
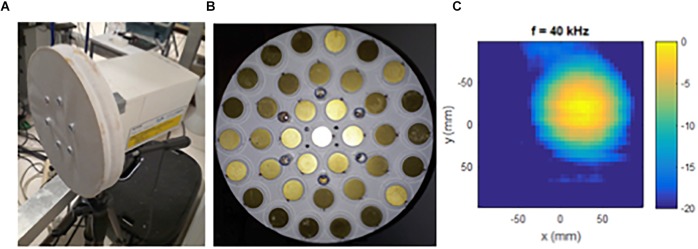
The non-contact vibrometer using airborne ultrasound (SONAR) to measure surface movements **(A)**, with front view of the transmission reception aperture **(B)**, and illustration of the ultrasonic emission reception focusing pattern at 40 kHz **(C)**.

The instrument (Figure 1A) was designed and built in-house (Institut Langevin, CNRS UMR 7587, Ecole Supérieure de Physique et Chimie Industrielles, Paris, France). The transmission reception aperture of the SONAR is composed of 37 piezoelectric diaphragms (Murata 7BB-20-6, Murata electronics, Tokyo, Japan) and of 6 high frequency microphones (Knowles FG-23329, Knowles Electronics, LLC. Itasca, IL, United States). As shown in Figure 1B, all the components are uniformly disposed on a hemispherical cup of 150 cm radius machined in PVC foam. The emitted ultrasounds then focus at 150 cm distance. The cup diameter is equal to 210 mm. All piezoelectric diaphragms are used for emission. They are connected in parallel and driven by a unique electronic amplifier with a voltage range from -20 to +20 V. The microphones are all connected in a parallel configuration. The resulting unique microphone signal is 40 db amplified in the operational bandwidth 20–60 kHz. Emission and reception signals are directed toward a numeric acquisition board (Agilent U2542A, Agilent technologies, Santa Clara, CA, United States). The sampling frequency for both the emission and reception signals is 320 kHz.

The working principle of the SONAR system is as follows. A frequency modulated ultrasonic wave is periodically emitted toward the subject to study. The emission period is 1/75 s. Thereafter the echo reflected waves are detected by the microphones. A cross-correlation is computed between two successive echo reflected signals and the time delay is determined. This time delay is proportional to the ratio of the differential displacement of the monitored surface by the ultrasound propagation velocity (∼ 345 m/s). In the end, the SONAR system measures the normal velocity of the surface motion. The normal velocity measurement is averaged over a circular surface of approximately 110 mm diameter as shown by the ultrasonic emission reception focusing pattern in Figure 1C. The normal displacement of the surface motion is obtained by integrating the normal velocity data over time.

### Experimental Conditions and Protocol

The subjects were studied while sitting on a chair with their back straight and their neck in a neutral position. They were asked not to move during the recordings.

During the recordings, the front aperture of the SONAR was positioned either in front of the subjects at a 150 cm ± 10 cm distance, with the device mounted on a photographic tripod allowing adjustments of height and direction. A weak intensity laser beam mounted in the SONAR aperture and parallel to the SONAR beam was used to adjust the direction of the ultrasonic beam so that it was perpendicular to the measured surface.

Ventilatory data were collected over 1 min bouts of tidal breathing with the subjects breathing either through the PNT with a noseclip on (“SONAR-PNT” condition, all subjects) or breathing without the PNT (“SONAR-only” condition, 17 subjects). “SONAR-PNT” and “SONAR-only” acquisitions were performed “anteriorly,” namely with the SONAR positioned in front of the subjects and the ultrasonic beam focused on the xiphoid process (FRONT acquisitions). PNT-SONAR acquisition were also performed “posteriorly,” namely with the SONAR positioned behind the subjects, at a 45° angle, and the ultrasonic beam focused on the lower portion of the costal margin (BACK acquisitions). All these sequences were randomly performed.

### Statistical Analysis

Data distributions were checked for normality with Shapiro–Wilk’s test. All variables were not normally distributed and thus were described in terms of their median and interquartile range (Q1–Q3). They were also described in terms of their variability as assessed by their breath-by-breath coefficient of variation (CV).

The interchangeability of the PNT and SONAR measurements For each variable (Bf, TI, TE, TT, VT, and displacement) was tested by comparing the PNT (as gold-standard) and SONAR signals during the SONAR-PNT condition with the Passing-Bablok non-parametric regression (Passing and Bablok, 1983), under both FRONT and BACK conditions. Interchangeability was confirmed when 95% CI for intercept includes value « 0 » and when 95% CI for slope includes value « 1 ». Then, it can be concluded that there is no significant difference between obtained slope value and value one and there is no significant difference between SONAR and PNT measurements, so both methods can be used interchangeably.

In order to show whether or not an “observer and instrument effects” were present, the variability of SONAR-derived ventilatory measurements obtained during SONAR-only and during SONAR-PNT recordings was assessed through their coefficients of variation (CVs). For each breathing pattern variable, comparison was performed with Wilcoxon’s signed rank test. Wilcoxon’s signed rank test was also used to compare the “front” and “back” measurements under SONAR-only and SONAR-PNT conditions.

Although the study was not designed to assess the test-retest reliability, we compared the SONAR measurements obtained during the SONAR-PNT experiment with the SONAR measurements obtained during the SONAR-only experiment to have at least a glimpse of reliability, by bearing in mind that the different settings would impact on the results. Therefore, the test-retest reliability of temporal variables measured with SONAR-PNT versus SONAR-only in FRONT condition (17 subjects) was determined by calculating the interclass correlation coefficients (ICCs) and their associated 95% confidence intervals (95% CIs). The sum of the squares needed for the calculation of the ICC was obtained by using a two-way ANOVA mixed-effects model (Koo and Li, 2016). For all comparisons, a value of p below 0.05 was considered statistically significant. Statistical analyses were performed with GraphPad Prism 6.0 (GraphPad Software, Inc., La Jolla, CA, United States) and MedCalc Statistical Software version 14.8.1 (MedCalc Software bvba, Ostend, Belgium).

## Results

**FIGURE 2:**
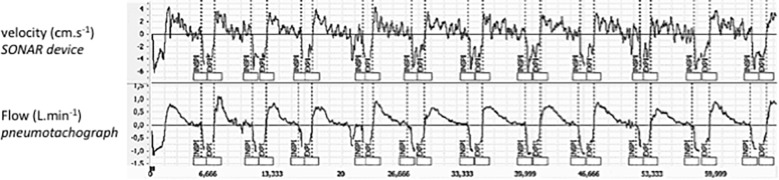
Examples of tracings, in one subject, of chest wall velocity as measured by the SONAR **(Top)** and of ventilatory flow as measured with the pneumotachograph **(Bottom)**. These recordings are obtained with the subject breathing through the pneumotachograph with a nose clip on (SONAR-PNT condition) and the SONAR in the anterior position (FRONT condition).

Figure 2 provides an example of the simultaneous recording of flow with the PNT and velocity of chest wall displacements with the SONAR. The two signals were expectedly slightly out of phase, with SONAR tending to run ahead of PNT but not in a systematic manner. This resulted in some degree of hysteresis on X-Y plots. On average the differences between the start of inspiration measured on SONAR and on PNT [0.01 s (-0.05 to 0.06)] and between the start of expiration measured on SONAR and on PNT median [I0.03 s (-0.03 to 0.05)] did not reach statistical significance (*p* = 0.86 and 0.39, respectively).

**FIGURE 3:**
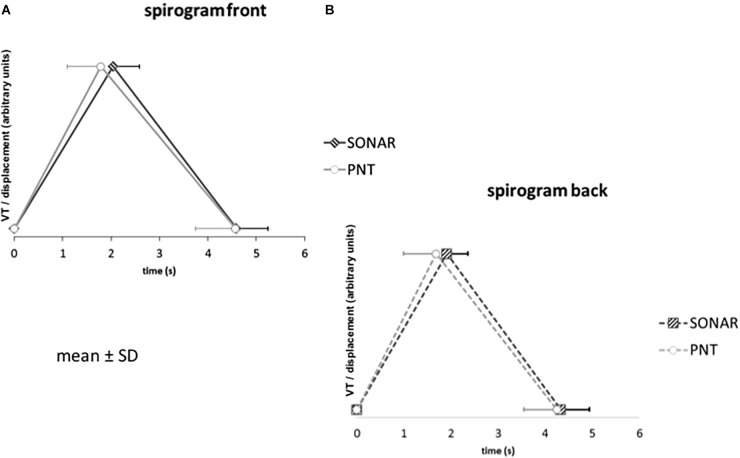
Average spirograms (with indications of mean and standard deviation) reconstructed from the pneumotachograph signal (PNT) and the non-contact vibrometer using airborne ultrasound (SONAR) in the whole study population, with the SONAR in front of the subjects **(A)** or in their back **(B)**. The *Y*-axis indicates tidal volume (VT) or SONAR-related tidal volume displacement (both in arbitrary units to be comparable).

**FIGURE 4:**
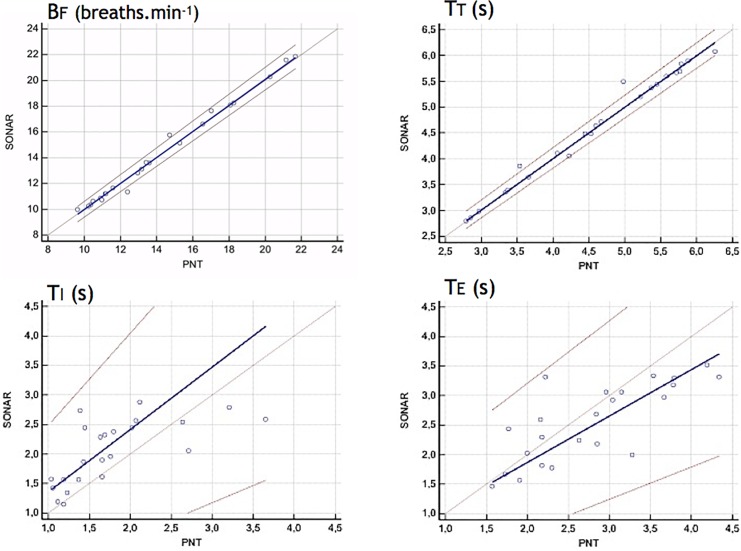
Comparison of discrete respiratory variables (Bf, breathing frequency -breaths min^-1^-; TT, breathing cycle period -s-; TI, inspiratory time -s-; TE, expiratory time -s-) derived from the pneumotachograph signal (PNT) and the non-contact vibrometer using airborne ultrasound (SONAR) with the SONAR device placed in front of the subjects. For these four variables, the Passing-Bablok regression analysis concluded to interchangeability between the two measurement methods (see section “Materials and Methods” for details). This was not the case for tidal volume vs. displacement insofar as these two variables have different meaning and dimensions.

**FIGURE 5:**
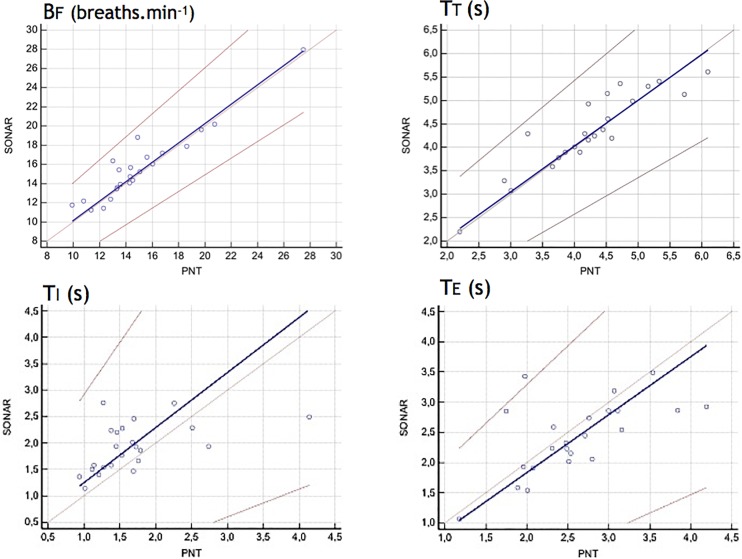
Comparison of discrete respiratory variables (Bf, breathing frequency -breaths min^-1^-; TT, breathing cycle period -s-; TI, inspiratory time -s-; TE, expiratory time -s-) derived from the pneumotachograph signal (PNT) and the non-contact vibrometer using airborne ultrasound (SONAR) with the SONAR device placed in the back of the subjects. This was not the case for tidal volume vs. displacement insofar as these two variables have different meaning and dimensions.

Table 1 provides the ventilatory variables measured with the PNT and the SONAR under the SONAR-PNT condition, during FRONT recordings (see also Figure 3). For BF, TT, TI, and TE the linearity condition to apply the Passing-Bablok regression was met, and the criteria for interchangeability were also met (Table 2 and Figure 4, 5). Expectedly, even though the relationship between VT and displacement was linear and the correlation between them strong, these variables did not meet the criteria for interchangeability (Table 2). The exact same conclusions were reached regarding the BACK recordings (Table 3).

**Table 1:** Ventilatory variables measured with the pneumotachograph (PNT) and the non-contact vibrometer using airborne ultrasound (SONAR) during synchronous recordings of the two datasets (PNT-SONAR condition, FRONT recordings).

SONAR-PNT condition	BF (breath min^-1^)	TT S	TI S	TE S	VT L	Displacement cm
PNT	13.2 (11.1–16.8)	4.6 (3.6–5.5)	1.7 (1.3–2.0)	2.8 (2.2–3.4)	0.7 (0.5–0.8)	–
SONAR	13.1 (11.0–17.1)	4.6 (3.7–5.5)	2.0 (1.6–2.5)	2.6 (2.0–3.1)	–	3.0 (2.4–4.1)
SONAR-PNT condition	CV BF	CV TT	CV TI	CV TE	CV VT	CV displacement
PNT	0.10 (0.08–0.13)	0.10 (0.09–0.12)	0.10 (0.08–0.12)	0.13 (0.11–0.17)	0.15 (0.11–0.24)	–
SONAR	0.10 (0.08–0.12)	0.10 (0.08–0.12)	0.12 (0.10–0.15)	0.13 (0.10–0.16)	–	0.17 (0.14–0.21)


*Data are presented as median (Q1–Q3). BF, breathing frequency; TT, breathing cycle period; TI, inspiratory time; TE, expiratory time; VT, tidal volume; s, second; L, liter; CV, coefficient of variation.*

**Table 2:** Summary of Passing-Bablok comparisons of the ventilatory variables measured with the pneumotachograph (PNT) and the non-contact vibrometer using airborne ultrasound (SONAR) during synchronous recordings of the two datasets (PNT-SONAR condition, FRONT recordings).

SONAR-PNT condition FRONT	BF (breath min^-1^)	TT S	TI S	TE S	VT/displacement L cm
Intercept A	0.12	-0.04	0.30	0.30	-9.63
95% CI	(-0.53 to 0.14)	(-0.17 to 0.03)	(-0.44 to 0.97)	(-0.41 to 1.09)	
Slope B	1.01	1.01	1.05	0.78	18.86
95% CI	(0.99–1.04)	(0.99–1.04)	(0.56–1.46)	(0.55–1.06)	
*p*-Value	*p* = 0.78	*p* = 0.42	*p* = 0.42	*p* = 0.99	*p* = 0.97

**SONAR-PNT condition FRONT**	**CV BF**	**CV TT**	**CV TI**	**CV TE**	**CV VT/CV displacement**

Intercept A	0	0	0.01	0.01	0.12
95% CI	(-0.01 to 0.03)	(-0.01 to 0.01)	(-0.06 to 0.08)	(-0.05 to 0.04)	(0.01–0.17)
Slope B	1	1	1	1	0.4
95% CI	(0.75–1.13)	(0.88–1.14)	(0.50–1.67)	(0.78–1.44)	(0.00–1.00)
*p*-Value	*p* = 0.98	*p* = 0.74	*p* = 0.72	*p* = 0.45	*p* = 0.26


*BF, breathing frequency; TT, breathing cycle period; TI, inspiratory time; TE, expiratory time; VT, tidal volume; s, second; L, liter; CV, coefficient of variation.*

**Table 3:** Summary of Passing-Bablok comparisons of the ventilatory variables measured with the pneumotachograph (PNT) and the non-contact vibrometer using airborne ultrasound (SONAR) during synchronous recordings of the two datasets (PNT-SONAR condition, BACK recordings).

SONAR-PNT condition BACK	BF (breath min^-1^)	TT S	TI S	TE S	VT/displacement L cm
Intercept A	0.07	0.13	0.20	0.09	-1.03
95% CI	(-2.53 to 2.09)	(-0.56 to 0.87)	(-0.99 to 0.95)	(-0.97 to 0.72)	(-5.21 to 0.14)
Slope B	1.01	0.97	1.04	0.96	3.00
95% CI	(0.87–1.20)	(0.78–1.14)	(0.53–1.97)	(0.61–1.28)	(1.02–10.00)
*p*-Value	*p* = 0.78	*p* = 0.18	*p* = 0.78	*p* = 0.99	*p* = 0.18


*BF, breathing frequency; TT, breathing cycle period; TI, inspiratory time; TE, expiratory time; VT, tidal volume; s, second; L, liter.*

In 17 subjects, SONAR recordings were repeated (with the SONAR in front of the subjects) while the subjects breathed freely, without the PNT. Table 4 provides the ventilatory variables derived from the SONAR signals in the two conditions (SONAR-PNT and SONAR-only). Table 5 provides the corresponding coefficients of variations. No significant difference was detected between SONAR-PNT variables and SONAR-only variables, both in absolute terms and in terms of their variability.

**Table 4:** Ventilatory variables measured with the non-contact vibrometer using airborne ultrasound during synchronous pneumotachograph-SONAR recordings (PNT-SONAR) and with the SONAR only (SONAR-only).

SONAR signal	BF (breath min^-1^)	TT S	TI S	TE S	Displacement cm
In SONAR-PNT condition	13.1 (11.2–17.6)	4.6 (3.6–5.5)	1.9 (1.6–2.4)	2.7 (2.0–3.1)	3.0 (2.3–5.3)
In SONAR-only condition	15.7 (13.3–17.4)	4.0 (3.5–4.7)	1.7 (1.4–2.2)	2.1 (1.9–2.8)	2.4 (2.3–2.5)
*p*-Value	0.12	0.07	0.13	0.06	0.10


*Data are presented as median (Q1–Q3). BF, breathing frequency; TT, breathing cycle period; TI, inspiratory time; TE, expiratory time; s, second.*

**Table 5:** Coefficients of variations of the ventilatory variables measured with the non-contact vibrometer using airborne ultrasound during synchronous pneumotachograph-SONAR recordings (SONAR-PNT) and with the SONAR only (SONAR-only).

SONAR signal	TT S	TI S	TE S	Displacement cm
In SONAR-PNT condition	0.09 (0.08–0.12)	0.12 (0.10–0.16)	0.11 (0.10–0.16)	0.18 (0.15–0.22)
In SONAR-only condition	0.11 (0.08–0.15)	0.15 (0.14–0.16)	0.14 (0.11–0.22)	0.23 (0.16–0.29)
*p*-Value	0.74	0.18	0.52	0.19


*Data are presented as median (Q1–Q3). TT, breathing cycle period; TI, inspiratory time; TE, expiratory time; s, second.*

Test-retest reliability of the temporal ventilatory variables (TI, TE, TT) in the conditions PNT-SONAR and SONAR-only in FRONT condition are presented in Table 6. ICCs indicated moderate reliability with the exception of Expiratory time (TE) measurements that were judged unreliable because of an interval confidence crossing value « 0 ».

**Table 6:** Interclass correlation coefficients (ICCs) and their associated 95% confidence intervals (95% CIs) between the SONAR measurements in FRONT condition obtained during the SONAR-PNT experiment vs. the SONAR measurements obtained during the SONAR-only experiment in 17 subjects.

SONAR signal	TT S	TI S	TE S
ICC (95% CI)	0.66 (0.12–0.87)	0.70 (0.22–0.90)	0.60 (-0.02 to 0.85)


*TT, breathing cycle period; TI, inspiratory time; TE, expiratory time; s, second.*

Statistical differences (*p* < 0.05) were found regarding TI and TT during the SONAR-only experiments in FRONT vs. BACK conditions and during the PNT-SONAR experiments in FRONT vs. BACK conditions.

## Discussion

Firstly, this study validates the non-contact vibrometry using airborne ultrasound (SONAR) as a technique to describe the resting breathing pattern of healthy humans. Although the chest velocity signal derived from the SONAR recordings and the airway opening flow signal derived from the PNT recordings were, expectedly, not identical (Figure 2), they gave access to discrete ventilatory variables (BF, TT, TI, TE) that were interchangeable (Figure 3–5 and Tables 1–3). Secondly, the study shows that the non-contact vibrometer using airborne ultrasound cannot only provide an accurate description of breathing pattern with the measuring device placed “traditionally” in front of the subject, but also when it is placed in the back of the subject. This is of particular importance regarding applications where it would be crucial that the participants be unaware of their breathing being measured.

### Comparison With Existing Data

Using an ultrasonic contactless sensor to measure breathing has already been proposed (Arlotto et al., 2014) but with a very different approach from the one used in this study. Arlotto et al. (2014) proposed to use a technology resembling the present one to measure the frequency shifts produced by the difference in velocity between exhaled air flow and the ambient environment. In our study, the SONAR approach was used to image the displacements of the surface of the chest wall. The two approaches have different objectives and different outputs. Recently, several tools using ultrasonic sensors to measure breathing rates have been described (Wang et al., 2017; Dang et al., 2018). The corresponding studies suggest that the technology is adequate but these studies did not provide extensive validation data. Our study confirms that ultrasonic approaches to measure breathing are of value. It does so through a validation process that relies both on the reference to a gold standard of physiological respiratory measurements and on a large number of subjects studied in a variety of conditions. Of note, other contactless breathing monitoring systems have been described. Structured light plethysmography is the most advanced one regarding validation against reference measurements (Elshafie et al., 2016; Hmeidi et al., 2017; Motamedi-Fakhr et al., 2017a,b; Nierat et al., 2017). It, however, requires visible illumination of the subject, which prevents its use in a stealthy context. It also prevents its use during sleep studies where obscurity is required by definition. The use of radiofrequencies has also been proposed to monitor cardiac and respiratory activities. Data have been provided using radar based schemes (Droitcour et al., 2009a,b; Salm and Molisch, 2011; Adib et al., 2015; Gennarelli et al., 2016; Nguyen et al., 2016) or wifi based schemes (Abdelnasser et al., 2015; Liu et al., 2015; Yang et al., 2016), but radiofrequency based signals are easily disrupted by environmental interferences. Finally, video-based respiratory monitoring has also been the object of several studies (Janssen et al., 2016; Wijenayake and Park, 2017), as well as time-of-flight sensors (Schaller et al., 2008; Sharp et al., 2017). All these technological investigation emphasize the importance of breathing monitoring under ecological conditions.

### Strengths and Limitations

Although the recording time was short, our study provides extensive physiological validation of non-contact vibrometry using airborne ultrasound to measure breathing in humans. This validation is obtained against a measurement technique widely used in physiological studies and that is considered as one of the gold standard. It derives from a statistical approach that has been specifically designed to test the interchangeability of two linearly related measures of the same thing, which was the case here. It pertains to various aspects of the physiological signal, namely the absolute values of discrete variables extracted from this signal and their breath-by-breath variability. In addition, it is seemingly the first time that it is demonstrated that a contactless device placed in the back of the subject can precisely describe the breathing activity of this subject. Our results should therefore be useful to the medical community, by allaying concerns about the validity of ultrasound derived measurements of breathing and avoiding the need to repeat the process for each new device in development.

From a methodological point of view, the feasibility and intra-observer and inter-observer reliability (i.e., reproducibility) of the SONAR measurements were not investigated in this study. Further investigation designed to specifically answer these questions is welcome in the future.

From a technical point of view, our device is currently limited (1) by the necessity to maintain a constant distance between the SONAR aperture and the subject and (2) by the recording time (1 min only). The first point should easily be alleviated by coupling the device to a motion sensor and dynamically adapting the focus of the SONAR according to the data provided by the subject. The recording time will be substantially increased in the next model of SONAR.

We expected to find differences between the recordings performed under the PNT-SONAR condition with the SONAR recordings performed under the SONAR-only condition, according to the instrumental observer effect principle. Even though such differences existed, both in the values of the respiratory variables themselves and in their variability, they did not reach the threshold of statistical significance (Tables 4, 5). This is not consistent with previous results (Fiamma et al., 2007a; Motamedi-Fakhr et al., 2017a) and could be viewed as questioning the very validity of contactless measurements. However, the subjects who participated in our study were well aware that their breathing was being measured, and they were instructed to sit straight in the chair used during the experiments, to keep their own back in contact with the chair back, and to avoid body movements. These elements might have been sufficient to induce an observer effect attenuating the differences between the two recording conditions. We acknowledge that this prevents us from evaluating the value of the SONAR device for real life measures and also prevents us to verify if this approach can eliminate the cognitive observer effect. This was, however, not the objective of the study, and further work will be necessary to formally test these points. In a similar study pertaining to structured light plethysmography, we have shown that breathing through a pneumotachograph was sufficient to significantly modify breathing variability (Nierat et al., 2017).

## Perspectives and Conclusion

Validating a non-contact vibrometry using airborne ultrasound device to measure ventilatory breathing pattern, as achieved by this study, opens new perspectives. Such non-contact measurements can be performed in any ambient light condition: this could be useful in a variety of clinical environments for simplified respiratory monitoring. Examples include sleep laboratories for the diagnostic and quantification of sleep-related respiratory abnormalities; emergency departments, where the monitoring of respiratory frequency is currently more difficult and therefore less often performed than the monitoring of other vital signs (of note, monitoring respiratory frequency is particularly relevant to the safety of opioid treatments); telemedicine, where the use of respiratory monitoring is currently limited by the necessity for the patient to actively interact with a device to transmit relevant information. The same is true for measurements of breathing activity performed in non-medical contexts, for example, in the field of well-being oriented smartphone applications. The ability to measure breathing not only without equipping the subject of the patient with a device, but also to measure breathing unbeknown to the subject is of paramount importance in neurophysiology and psychophysiology studies. To date, in the absence of this possibility, it is for example very difficult to gauge the impact of emotional stimulations on breathing, or to evaluate the impact of non-automatic ventilatory commands on tidal breathing because the corresponding experiment almost unavoidably focuses the attention of the subjects of their respiratory system. One example of such a situation can be found in a recent study investigating the effects of observing the dyspnea of others in normal individuals (Herzog et al., 2018). This study reported that participants looking at pictures or videos of persons experiencing dyspnea reported dyspnea in themselves and negative affective feelings. These subjective experiences were corroborated by the presence of specific brain potentials. Yet respiratory measurements performed with a pneumotachograph did not detect changes in breathing pattern, which is surprising. This could have been due to the changes induced by the respiratory measurement apparatus being of greater magnitude than the changes induced by viewing the dyspneic pictures. Finally, the SONAR approach could be particularly practical to measure breathing easily in animal experiments. We therefore believe that non-contact vibrometry using airborne ultrasound will allow advances in these various fields.

## New and Noteworthy

Non-contact vibrometry using airborne ultrasound is a valid tool to measure thoracic displacements during resting breathing in normal humans. It gives access to an accurate description of breathing pattern and of its variability and could be used in true ecological conditions (devoid of observer effect due to instrumentation or subjects’ awareness).

## Data Availability

The datasets for this manuscript are not publicly available because datasets used and/or analyzed during the current study are private and available from the corresponding author only on reasonable request. Requests to access the datasets should be directed to thomas.similowski@upmc.fr.

## Ethics Statement

This study was carried out in accordance with the recommendations of the legal and ethical French authorities (Comité de Protection des Personnes Ile-de-France VI Pitié-Salpêtrière, Paris, France; ID-RCB 2017-A03665-48) with written informed consent from all subjects. All subjects gave written informed consent in accordance with the Declaration of Helsinki. The protocol was approved by the legal and ethical French authorities (Comité de Protection des Personnes Ile-de-France VI Pitié-Salpêtrière, Paris, France).

## Author Contributions

TS and R-KI conceived of the presented idea. M-CN and R-KI conceived and planned the experiments. R-KI developed the ultrasonic device. M-CN, PS, and R-KI carried out the experiments. M-CN, PL, B-PD, and R-KI processed the experimental data and analyzed the data. All authors provided the critical feedback and helped to shape the research, analysis, interpretation, and manuscript under the lead of TS.

## Conflict of Interest Statement

The authors declare that the research was conducted in the absence of any commercial or financial relationships that could be construed as a potential conflict of interest.
